# Gallbladder Mysteries: A Diagnostic Dilemma in Mirizzi Syndrome With Acalculous Presentation

**DOI:** 10.7759/cureus.46997

**Published:** 2023-10-13

**Authors:** Gavitha N Thabrew Wijeratne, Samadhi T Wijeratne, Nabila N Anika, Yusra H Hamid, Javeria Naz

**Affiliations:** 1 Medicine, Ross University School of Medicine, Miramar, USA; 2 Medicine and Surgery, Holy Family Red Crescent Medical College and Hospital, Dhaka, BGD; 3 Community Medicine, Faculty of Medicine, University of Khartoum, Khartoum, SDN; 4 Internal Medicine, Jinnah Sindh Medical University, Karachi, PAK

**Keywords:** chronic gallbladder stones, abstract, acalculous presentation, mirizzi syndrome, diagnostic dilemma, gallbladder mysteries

## Abstract

Mirizzi syndrome (MS) is an uncommon condition caused by chronic gallbladder stones, leading to external compression and obstruction of the common hepatic duct. This report details an unusual MS case in a 65-year-old man who experienced right upper abdominal pain, jaundice, fever, and nausea. Diagnostic tests, such as ultrasound and CT scan, indicated acute acalculous cholecystitis resembling MS. However, a magnetic resonance cholangiopancreatography (MRCP) confirmed no gallstones in the biliary system. The patient's laparoscopic cholecystectomy was successful, with tissue analysis revealing intense gallbladder inflammation and epithelial necrosis but no gallstones. This case emphasizes the diagnostic complexities of atypical MS presentations and the need for comprehensive diagnostic methods, including MRCP. Additionally, the report advocates for standardized terminology in medical literature to ensure clear communication among medical professionals.

## Introduction

Mirizzi syndrome (MS), an uncommon consequence of prolonged gallbladder stones, was first identified in 1948 [1]. This condition is triggered by the presence of a gallbladder neck stone or a cystic duct stone, which, with or without a cholecystocholedochal fistula, exerts pressure on the common hepatic duct [2]. In the United States, approximately 20 million people are afflicted by cholelithiasis, and MS is an infrequent complication, affecting approximately 0.1% of these individuals [3].

In the subhepatic region, severe inflammation and adhesions often affect the hepatoduodenal ligament, disrupting the typical anatomical relationships and dimensions. Acute acalculous cholecystitis (AAC) can lead to a syndrome that closely resembles multiple sclerosis in clinical progression and imaging characteristics [4]. This mechanism mirrors that of MS, where an external bile duct compression occurs due to a stone blocking the gallbladder neck or cystic duct. The literature has recorded very few instances of this particular complication of acalculous cholecystitis [5].

## Case presentation

A 65-year-old man presented to the outpatient department with a 10-day history of right upper abdominal pain, which was followed by jaundice, fever, and nausea over the last four days. He had no history of diabetes, hypertension, or viral hepatitis. Initially healthy, he suddenly experienced intense, dull pain in the right upper abdomen that radiated to the mid-back, alleviated by painkillers. He later developed a fever and noticed his eyes turning yellow. He also mentioned experiencing constipation for the past three days and having no prior health concerns or notable family medical history.

Diagnostic tests revealed markedly elevated total, conjugated, and unconjugated bilirubin levels, indicative of obstructive jaundice. Additionally, there were elevated white blood cell counts and neutrophilia, suggesting an underlying inflammatory process. The marked elevation in alkaline phosphatase (ALP) further pointed toward obstructive jaundice. The complete blood workup that was carried out initially is detailed in Table 1.

**Table 1:** Complete blood workup of patients showing many derangements. INR: international normalized ratio, APTT: activated partial thromboplastin time, WBC count: white blood cell count, RBC: red blood cells, HCT: hematocrit, MCV: mean corpuscular volume, MCH: mean corpuscular hemoglobin, MCHC: mean corpuscular hemoglobin concentration, ALT: alanine transaminase, AST: aspartate aminotransferase, ALP: alkaline phosphatase, ESR: erythrocyte sedimentation rate.

Coagulation Profile		
Tests	Results	Reference range
Prothrombin time-control	12	10-14 seconds
Prothrombin time-patient	09	Up to 13 seconds
INR	1.1	0.9-1.3
Control time	27	25-35 seconds
APTT	25	Up to 31 seconds
Hemogram		
WBC count	22.0	4-11 x10^9^/L
Total RBC	4.51	3.8-5.2 x10^12^/l
Hemoglobin	13.6	13-18 (g/dL)
HCT	39.5	35-46%
MCV	87.7	77-95 fl
MCH	30.2	26-32 (pg)
MCHC	34.5	32-36 (g/dL)
Platelets	108	150-400 x10^9^/L
Neutrophils	89	40-80%
Lymphocytes	06	20-40%
Monocytes	03	2-10%
Eosinophils	02	1-6%
Renal function tests		
Urea	235	10-50 mg/dl
Serum creatinine	5.5	0.5-0.9 mg/dl
Liver function tests		
Bilirubin total	29.0	0.3-1.2 mg/dl
S. conjugated bilirubin	28.6	<0.5mg/dl
S. unconjugated bilirubin	17.2	0.1-1.0mg/dl
Total protein	6.5	5.7-8.2 g/dl
Albumin	3.5	3.2-4.8 g/dl
ALT	230	Up to 40 U/L
AST	195	Up to 40 U/L
ALP	1325	40-120 U/L
Serum electrolytes		
Sodium	140	135-145 mmol/L
Potassium	4.8	3.5-5 mmol/L
Chloride	101	98-107 mmol/L
Calcium	8.6	8.5-10.5 mg/dl
Inflammatory markers		
ESR	83	0-25 mm/1^st^ hour

Following the initial findings, a more detailed assessment was conducted. An ultrasound revealed a rough liver texture and an enlarged common bile duct (CBD) without detectable stones. A positive Murphy's sign was noted, along with dilated intra- and extrahepatic biliary channels and an inconsistent liver texture. Acute acalculous cholecystitis was the initial diagnosis. However, certain clinical symptoms and ultrasound results hinted at an obstructive issue, leading to a CT scan for further clarity.

The CT scan showed mild CBD enlargement with a sudden constriction near the supraduodenal area, causing dilation in the intrahepatic biliary channels. No significant mass or radiodense stone was detected in the CBD, and there was no sign of any ampullary or periampullary mass. These CT results confirmed the obstructive nature of the pathology. An ERCP was then conducted to solidify the diagnosis and determine the best course of action.

**Figure 1:**
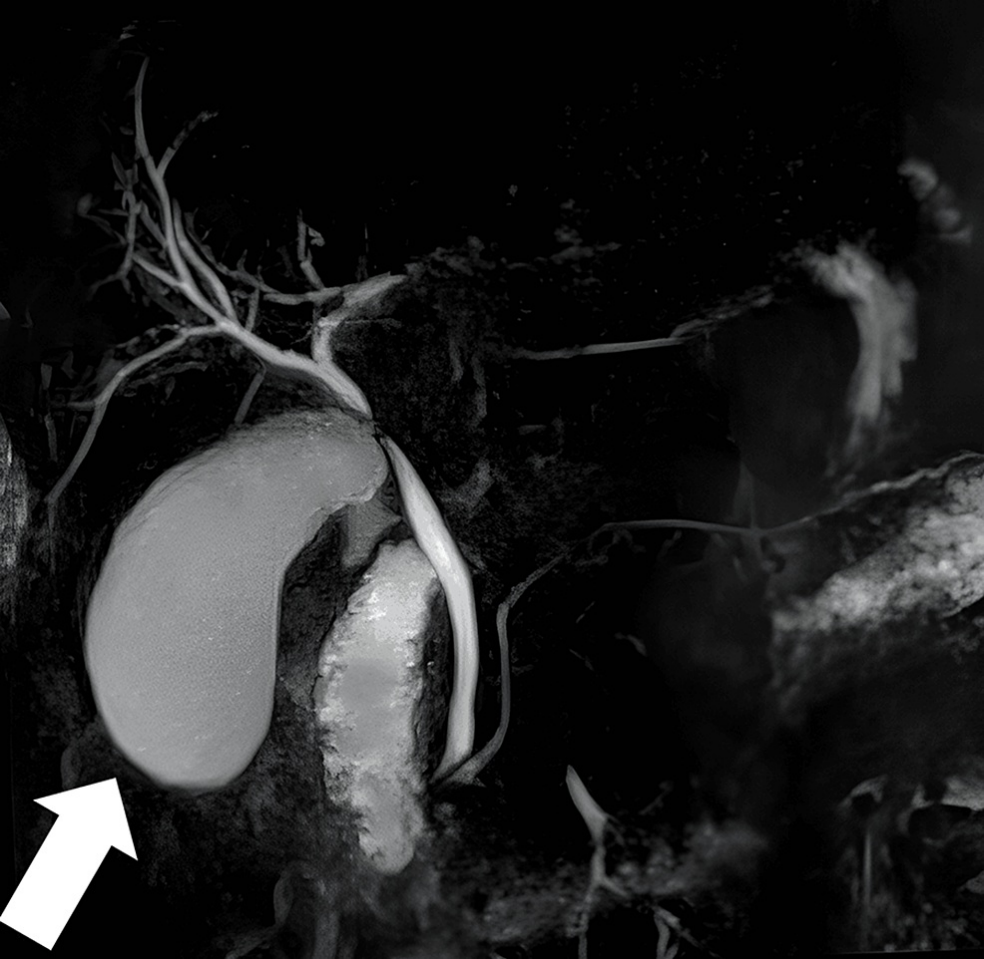
Magnetic resonance cholangiopancreatography (MRCP) depicts that there is external pressure on the common hepatic/common bile duct caused by the inflammation and enlargement of the gallbladder, with no detectable stones within the biliary system.

During ERCP, proximal duct dilation and strictures were identified, resulting in distal CBD obstruction, without apparent mass. Notably, the CBD exhibited wall damage. These ERCP findings guided subsequent surgical planning. Magnetic resonance cholangiopancreatography (MRCP) revealed gallbladder enlargement with wall thickening, causing extrinsic compression of the CBD, with no discernible stones within the biliary system (Figure 1).

A laparoscopic cholecystectomy was scheduled to alleviate the patient's symptoms. Intraoperatively, a significantly enlarged and edematous gallbladder was observed. Histopathological examination of the excised gallbladder showed marked signs of severe acute inflammation and epithelial necrosis, notably without the presence of gallstones in the gallbladder or cystic duct. Additionally, no evidence of carcinoma was detected.

After the surgery, the patient was advised on post-operative care, which included limited physical activity and a low-fat diet. The surgery's success was evident from the decreased ALP, AST, and ALT levels within a week. There were no post-operative complications. The patient is now closely monitored with regular check-ups to track long-term results and prognosis.

## Discussion

A common hepatic duct obstruction brought on by the extrinsic compression of an impacted stone in the cystic duct or Hartmann's pouch defines the unusual illness known as MS. It appears in about 0.35% of surgical cholecystectomies [6]. There may be a slight tendency for females and older populations, but recent studies have shown no male or female predilection [3]. More than 25% of MS patients are at risk for gallbladder cancer [7].

An enlarged and inflamed gallbladder can externally compress the common hepatic or bile duct, leading to jaundice and exacerbating AAC. This mechanism is similar to MS when a stone blocks the gallbladder neck or cystic duct, causing external bile duct compression. Ippolito first reported this syndrome in 1993 using ultrasound (US) and endoscopic retrograde cholangiopancreatography (ERCP) [8]. The study detailed a young male patient with recurring discomfort in the right upper abdomen and jaundice. However, the biliary obstruction was not due to AAC but was caused by an exceptionally swollen "congestive" gallbladder resulting from "cystic duct syndrome" [5].

It is noteworthy to note that there is disagreement on the nomenclature used to define this illness. These three published case reports each had a different terminology employed by the writers. The ailment was referred to as "acute acalculous cholecystitis associated with common hepatic duct obstruction: a variant of Mirizzi's syndrome" in Ippolito's paper [8]. While Ahlawat [9] used the title "Acute acalculous cholecystitis simulating MS: a very rare condition," Mergener et al. titled their research "Pseudo-Mirizzi syndrome in acute cholecystitis" [10]. We think that the aforementioned entity qualifies as a particular subtype of MS [5].

In acalculous cholecystitis cases, the diagnosis of MS is often made using percutaneous transhepatic cholangiography, US, or ERCP [8-10]. To treat jaundice, choledochal stenting was initially employed [10]. All patients underwent surgery: two had open cholecystectomy [8,9], and one had laparoscopic cholecystectomy [10]. Each of the three AAC cases showed gangrenous inflammation in the gallbladder [8-10]. Despite not having biliary drainage in all cases (only one of the three patients had it implanted) [5], jaundice and other symptoms improved.

In patients presenting with symptoms suggestive of MS, particularly those manifesting jaundice, right upper abdominal pain, and imaging findings indicating biliary obstruction, a comprehensive differential diagnosis should be meticulously considered. Conditions such as cholangiocarcinoma, choledochal cysts, primary sclerosing cholangitis, and impacted stones in the common bile duct are prominent differentials. Each of these conditions can manifest with overlapping clinical and radiological features, further complicating the diagnostic evaluation. Notably, gallbladder pancreatic heterotopia stands out as a rare differential. This involves the ectopic location of pancreatic tissue within the gallbladder, independent of anatomical and vascular links to the primary pancreas. Although typically asymptomatic, any inflammation or growth within this ectopic tissue can simulate cholecystitis or gallbladder tumors [11]. A thorough diagnostic workup, encompassing advanced imaging and, when required, histopathological examination, becomes indispensable in distinguishing MS from these conditions, ensuring precise clinical management.

MRCP in our case supported the diagnosis of MS in acalculous cholecystitis. Therefore, we believe that MRCP should be an effective technique to detect any type of MS, including this uncommon illness of acute acalculous cholecystitis determining MS, even though more research is required to draw a firm conclusion.

## Conclusions

MS is a rare gallbladder stone complication that poses diagnostic and management challenges. This report describes an uncommon MS case where acute acalculous cholecystitis mimicked clinical and radiological signs of MS. The MRCP-confirmed absence of gallstones in the biliary system added to the diagnostic complexity. The patient's successful laparoscopic cholecystectomy highlights the value of a thorough diagnostic approach, including MRCP, to distinguish MS-induced common hepatic duct obstruction from other causes. Clinicians should recognize this atypical MS presentation for prompt, appropriate intervention. The literature's varied terminologies for this condition indicate a need for standardized naming to enhance clarity and communication among medical professionals.
